# Integrating Psychotherapy Concepts: An Elective for Teaching Medical Students Through Observation and Applied Learning

**DOI:** 10.1007/s40596-025-02209-4

**Published:** 2025-10-08

**Authors:** Bria Williams, Josef Rivera, Annika Kisha, Alexandria Nasr, Allison Cowan, Brenda Roman, Bethany Harper, Larrilyn Grant

**Affiliations:** Wright State University Boonshoft School of Medicine, Dayton, OH USA

Psychotherapy has been a cornerstone of psychiatric treatment for over a hundred years [1] and remains a recommended treatment for multiple mental health conditions [2, 3]. Psychiatry residents must acquire proficiency in various psychotherapeutic modalities [4]. While skills learned administering psychotherapy are fundamental to psychiatrists, all clinicians may benefit from skills and insights gained through psychotherapy training, which can help navigate patient challenges such as resistance, treatment compliance, transference, and countertransference [5]. The Association of Directors of Medical Student Education in Psychiatry (ADMSEP) recommends all medical students gain a basic understanding of psychotherapy, including the ability to discuss common modalities and recommend appropriate therapies [6].

Despite recommendations, psychotherapy education in undergraduate medical education (UME) remains limited [5, 7, 8]. Several studies have utilized interactive methods—patient cases, group discussions, video clips, and role playing—to teach psychotherapy [9–12]. While these offer unique learning opportunities, students often lack direct patient exposure and the chance to apply their skills in practice.

In 2002, the Wright State University Boonshoft School of Medicine (WSU BSOM) launched a fourth-year elective to enhance clinical training. This report describes that elective, which aims to bridge the gap between theory and practice in psychotherapy education.

## Elective Overview

The 4-week elective is offered one month per year to fourth year medical students at the WSU BSOM. Due to high demand and limited availability, students are required to be interested in pursuing psychiatry residency and complete a two-prompt essay (please describe your interest in psychiatry and psychotherapy in 500 words or less) that is reviewed by faculty and staff. Five students are then selected for the elective.

The educational structure of this psychotherapy elective is built upon four key pillars: didactics, patient interactions, psychotherapy oversight, and the completion of a scholarly project. The elective is a total of 76 h of direct teaching and patient experiences, with 35 h devoted to elective didactics, 20 h dedicated to resident didactics, 4 h to patient experiences, 12 h to supervision, and 5 h to case conference. The remainder of the time allows for students to prepare for supervision, reflect on the patient experiences, write up the case, and research and complete their scholarly project.

### Didactics

Students participate in elective-specific didactics covering a diversity of topics from the foundations of psychotherapy to a more niche selection of discussions based on the interests of residents and attendings (Table 1). Students also attend psychotherapy didactics with residents on topics including psychotherapy case presentations, journal club, various therapeutic modalities, and how to combine psychotherapy with psychopharmacology.

**Table 1:** A list of foundational and unique topics taught over the years

**Foundational topics**
Essentials of Psychodynamic Psychotherapy
Essential Techniques for the Beginning Psychodynamic Psychotherapist
Cognitive Behavioral Therapy
Child and Adolescent Psychotherapy
Supportive Psychotherapy
Transference/Countertransference
**Unique topics**
Climate Aware Psychotherapy
Discussing ‘Taboo’ Topics of Clinical Importance
Humor in Therapy
Fable Fairytales and Spiderman Hero’s Journey
Evidence-Based Practices for Post Traumatic Stress Disorder
Harm Reduction Psychotherapy
MDMA Assisted Psychotherapy

#### Considerations for Implementation

In the early years of the course, the same faculty members were consistently recruited to teach specific didactic sessions. However, this approach led to challenges with scheduling and faculty availability. To address this, a repository of lecture materials was developed over time. Faculty members now select the topics they are most interested in or comfortable teaching and are provided with corresponding PowerPoint Presentations, which they can adapt for use during the academic year. This has helped recruit more faculty to engage in lectures and has avoided having to create lectures annually. Additionally, chief residents with a strong interest in education and psychotherapy have taken an active role in teaching. They are given a half-day of protected time to focus on educational and psychotherapy-related initiatives, which they have used to contribute to this course—helping to alleviate the faculty’s teaching load.

### Patient Interactions

Students are assigned one prospective patient during the first week of the elective. Students meet weekly with the patient via telehealth or in person for four weeks. The goals of these sessions are to complete a thorough intake (description of the patient, chief complaint, history of present illness, past psychiatric history, biological factors, developmental and personal history, diagnosis) and to construct a detailed interpretation of the psychological factors impacting the patient (drive theory, ego defenses, Erikson stages, insight, psychological mindedness, object relations, self-psychology, transference/countertransference). Throughout the elective, this information is acquired and organized into a case conference document based on an adaptation by Sherry Hatcher, PhD, from Nancy William’s Psychoanalytic Case Formulation [13] that is presented to an attending physician in the presence of their medical student colleagues at the end of the elective. Before the elective started, patients agreed to have their appointments recorded and that a resident physician would be assigned to them after four appointments to begin psychotherapy treatment.

#### Considerations for Implementation

Patient selection is crucial for a successful elective. Patients must agree to a video-recorded intake with a medical student before transitioning to a resident for psychotherapy. These intake sessions are free, aiding recruitment. Screening is conducted by the psychotherapy chief resident and clinic director. Eligible patients are adults (18+) motivated for treatment with identifiable goals. Exclusion criteria include recent psychiatric hospitalization (within five years), severe mania history, active substance use disorder hindering engagement, or seeking medication management alone. Clinic staff inform patients about relevant policies and treatment agreements and encourage open communication with their providers. If a patient is unable to continue therapy, the provider initiates a conversation to understand the reason for discontinuation, explore any clinical or logistical barriers, and collaboratively determine next steps. This may include arranging appropriate follow-up care, transitioning to another provider, or identifying alternative treatment options.

A key challenge in implementing the elective is maintaining sufficient patient volume to support five additional learners, especially given that second- to fourth-year residents also require psychotherapy cases. Strategies to address this include expanding outreach efforts and scheduling the elective later in the academic year when more patients may be available. In situations where patient availability is limited, alternative teaching methods—such as live role-playing sessions between chief residents—can be used to reinforce core psychotherapy principles.

### Psychotherapy Oversight

Students discuss their patient cases weekly with an attending physician and medical student colleagues. These case presentations are conversational and informal in nature, allowing opportunity for clarifying questions, discussions on transference and countertransference, and reviewing video clips from the recorded session. Throughout psychotherapy oversight, both the student and colleagues better refine their conceptualization of the patient. Students receive feedback on aspects of the case that could be further developed, guidance on asking more nuanced questions, and insights into their interview styles through a review of the recorded videos. For students, this is a rare opportunity to have feedback on their questions and engagement with the patient, based on direct observation through the videos. The last week of the elective, students participate in a case conference, where they present a detailed case conceptualization of the patient and their interpretation of psychological factors relevant to their patient. This conceptualization is given to the resident physician, who is assuming responsibility for the ongoing psychotherapeutic relationship. Residents carefully review the case conceptualization prior to meeting with the patient, which allows them to enter treatment with a clearer framework and begin psychodynamic work more effectively.

#### Considerations for Implementation

The medical students are meeting with an attending faculty member each week to provide education on their case. The attending faculty member volunteers their time to provide oversight and education to students. This may limit the availability of some faculty members to partake in psychotherapy oversight and could potentially be remedied with grant funding. Grant funding may be located through State Departments of Higher Education, local seed grants (sometimes at individual institutions), and Educational Grants through the Association of American Medical Colleges, and the Association of Directors of Medical Student Education in Psychiatry website.

At the initiation of the elective in 2002, the attending faculty member was also partaking in all case conferences and leading most of the didactics, which further limited clinical productivity. This barrier has been addressed by recruiting multiple faculty members to engage in case conference and didactics. Additionally, the psychotherapy chief resident now aids in all key portions of the elective which reduces burden on faculty.

### Scholarly Project

Students were instructed to write a 2000–3500 word paper detailing a psychotherapy-related topic of the student’s choosing or writing a case/vignette description of a de-identified patient case that had relevant clinical pearls.

#### Considerations for Implementation

Throughout the elective, the total number of student publications has not been formally tracked or recorded. To ensure compliance, these regulatory bodies should be consulted at the outset, and students should be paired with a faculty mentor who is interested in supporting the publication of their scholarly projects.

## Student Perspectives and Outcomes

Due to changes in data collection procedures, only course evaluations from 2014 to 2023 were available for analysis to assess student satisfaction and educational outcomes. Evaluations used a 5-point Likert scale, with 5 indicating “strongly agree.” Of 38 students who completed the elective, 20 provided feedback. Descriptive statistics summarized key educational ratings, and qualitative feedback identified common themes related to strengths and areas for improvement. Students gave an average rating of 5.00 for communication, clinical problem-solving, and medical knowledge development. High ratings were also given for instruction usefulness, faculty and resident modeling of professionalism, and timely feedback. The lowest-rated items were receiving timely and constructive feedback on written work (4.67) and exposure to an adequate breadth and variety of clinical problems (4.67), though these remained within a high range. Students valued learning from multiple faculty and appreciated didactics on individual therapy modalities. The hands-on experience of managing their own patients was cited as essential in refining skills. Notably, 26% of students felt the elective influenced their residency search, emphasizing psychotherapy. Several students with the assistance of faculty published scholarly work in peer-reviewed journals [14–18]. Suggestions for improvement included discussing a broader range of therapies earlier in the rotation and considering a longitudinal rotation to follow patients over a year.

## Barriers and Lessons Learned

### Cost Considerations

Since medical students are unable to bill for patient care, this elective does not generate funding or productivity for faculty involved. While we have been fortunate to have faculty be able to volunteer some of their time, this may be remedied by applying for grant funding for this important hands-on experience for medical students.

### Timing

The time of year the elective is held is also important. At our institution, the elective is currently held in January. When it was held earlier in the academic year, it was difficult to have enough patients for students and residents due to the second-year resident class starting their outpatient therapy clinics and needing new patients. At the conclusion of the elective, another challenge was transferring these patients to residents whose schedules were already full with new patients. In January, some residents have completed therapy with patients or are ready to add more patients to their schedule so may start engaging with the transfers from this elective. Medical students have suggested this elective be longitudinal, which has not been feasible based on current staff support. Some medical students prefer this elective to not occur during interview season; however, January is typically at the end of the interview season and has not placed undue burden on interviews for residency interviews in our experience. Having medical students miss portions of the elective for residency interviews was an issue when the elective was held earlier in the year.

### Scheduling and Administration

Having a dedicated individual to create the schedule for the elective each year is a critical part for this elective to be successful. We currently have our medical student coordinator for psychiatry fill this role.

### Research and Dissemination

With medical education research growing in the past decades, we have begun to evaluate our courses more thoroughly. We recommend that before initiating a similar elective, one should carefully plan for research and obtain both Institutional Review Board (IRB) approval and appropriate permissions under the Family Educational Rights and Privacy Act (FERPA) early in the process is critical. Due to role transitions and changes in data collection practices over the years, we were only able to analyze course evaluations from 2015 to 2023, when consistent records became available. To avoid similar gaps in the future, programs should implement a clear plan for how data will be collected, securely stored, and maintained across administrative transitions. Developing well-constructed knowledge and attitude-based surveys in advance ensures that data collection aligns with the courses’ objectives and provides reliable measures of student learning and experience. Programs should also consider how residents are utilizing the case conceptualization and what changes they recommend as they begin the transfer process with these patients.

## Future Perspectives

Student feedback indicates strong satisfaction with the elective and increased interest in psychiatry, particularly in psychotherapy. To further assess the impact, we plan to implement pre- and post-elective surveys and follow students into residency. We also aim to involve resident oversight sessions to support continuity of care. As the elective expands amid limited faculty resources, we will explore adding interactive and role-playing elements to enhance learning. Future evaluations will include resident and patient perspectives, particularly regarding transitions to long-term therapy. We also seek grant funding to sustain and grow the elective.

In conclusion, face-to-face psychotherapy training over a 4-week elective is feasible. Despite challenges such as cost, faculty availability, and patient selection, we offer practical strategies to address these barriers. Future studies should assess long-term effects on both learners and patient care.

## Data Availability

The data that support the findings of this study are available from the corresponding author upon reasonable request.
